# Parvimonas micra bacteremia in a patient with colonic carcinoma

**DOI:** 10.22088/cjim.10.4.472

**Published:** 2019

**Authors:** Muhammad Shoaib Khan, Muhammad Ishaq, Mark Hinson, Bindu Potugari, Ateeq U Rehman

**Affiliations:** 1 Department of Internal Medicine, Marshfield Clinic Health System, Marshfield, Wisconsin, USA; 2 Department of Diagnostic Radiology, Marshfield Clinic Health System, Marshfield, Wisconsin, USA

**Keywords:** Parvimonas micra, Anaerobic bacteremia, Colonic carcinoma

## Abstract

**Background:**

*Parvimonas micra* is a gram-positive anaerobe and a part of the normal commensal flora of the gastrointestinal tract. Factors predisposing to anaerobic bacteremia include malignant neoplasms, periodontal disease, immune deficiencies, chronic renal insufficiency, decubitus ulcers and perforated abdominal viscus. Cases of Parvimonas bacteremia in a patient with esophageal carcinoma and in a patient following ERCP procedure have been reported but to our best knowledge no case has been reported yet in which a patient had colonic carcinoma.

**Case presentation:**

We present a rare case of a 94-year-old male who presented with chief complaint of fever and constipation. Complete blood count revealed normal white blood cell count anemia. Urinalysis came out to be unremarkable for any evidence of infection. Two blood cultures grew *Parvimonas micra* and *Gamella morbillorum* and patient was later switched to ampicillin-sulbactam as per blood culture susceptibility results. Echocardiogram came negative for any evidence of infective endocarditis. CT abdomen/pelvis showed soft tissue mass in the ascending colon just superior to the ileocecal valve (fig.1, 2). Colonoscopy showed non-obstructing eccentric mass (fig. 3). Biopsy of the mass revealed moderately differentiated adenocarcinoma. Because of lack of distant metastasis, surgical resection of the mass as definitive curative treatment was done.

**Conclusion:**

Immune deficiency is a risk factor for anaerobic bacteremia. Apart from immediately starting the patient on antibiotics, a thorough search for malignancy may be considered when a patient presents with anaerobic bacteremia, especially, when the source of infection is not known. Identifying malignancy in earliest stages may improve treatment outcome.

*Parvimonas micra* is a gram-positive anaerobic cocci and is a part of the normal commensal flora of the GIT (gastrointestinal tract). Factors predisposing to anaerobic bacteremia include periodontal disease, malignant neoplasms, immune deficiencies, chronic renal insufficiency, decubitus ulcers, perforated abdominal viscus and appendicitis. *Parvimonas micra* are highly vulnerable to antibiotic therapy and, therefore, antibiotic therapy may be started as early as possible without waiting for the culture results. Patients with the abovementioned anaerobic bacteremia predisposing factors should be worked up for malignancy in addition to identifying the source of infection. We present a case of anaerobic bacteremia for which early workup for malignancy revealed localized right sided colonic carcinoma. Patient was successfully cured with surgical resection.

## Case presentation

A 94-year-old male with past medical history of dyslipidemia, type 2 diabetes mellitus and hypertension presented with chief complaint of fever and weakness.

**Figure 1:**
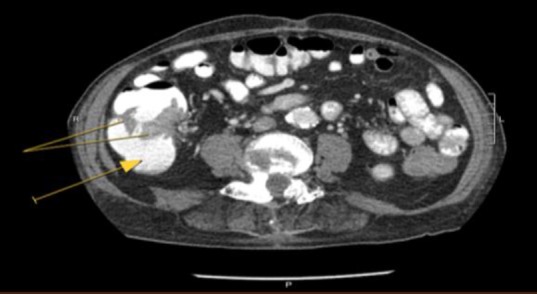
Axial view, CT scan abdomen with contrast: Solid arrow pointing at cecum. Solid lines pointing at contrast enhanced malignant mass. The mass is seen to be encircling more than 80 % of the circumference of the gut wall

**Figure 2:**
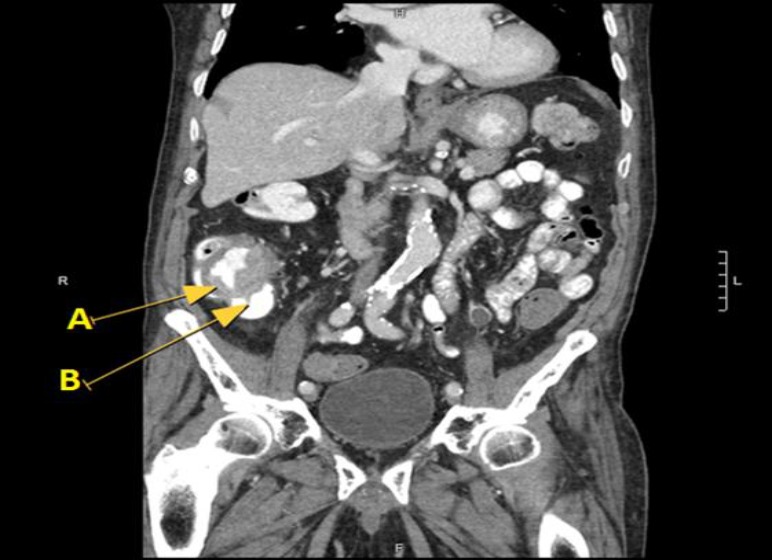
Coronal view, CT scan abdomen with contrast: Arrow ‘A’ pointing at malignant mass, which appears to encircle colonic wall to give an apple-core like lesion picture. Arrow ‘B’ is pointing at cecum

**Figure 3:**
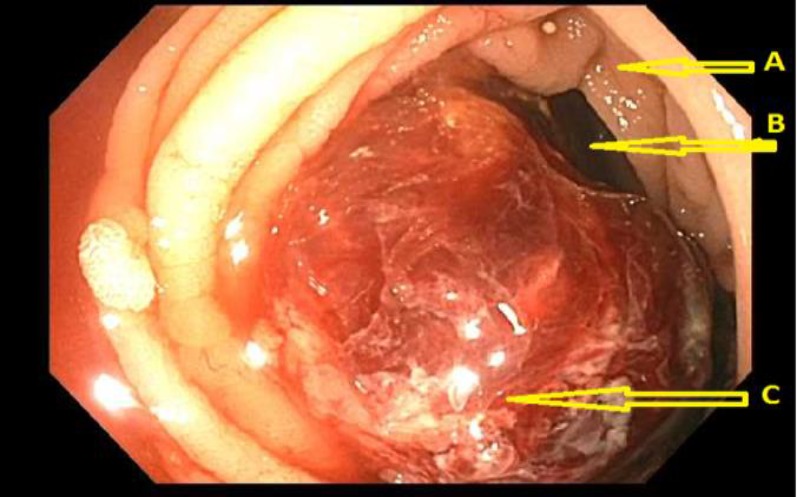
Colonoscopy: Arrow ‘A’ is pointing at colonic wall. Arrow ‘B’ is pointing at the lumen of the colonic wall. Note that lumen is severely narrowed by the malignant mass (pointed at by the arrow ‘C’). Arrow ‘C’ pointing at malignant mass, which appears to be eccentric in position and nearly occluding the lumen of the colon. Scattered areas of necrosis can also be seen on the malignant mass

Patient denied recent history of sore throat, ear pain, cough, diarrhea, urinary frequency or dysuria. On further inquiry, patient mentioned that he had constipation and had to strain a lot to pass stools. Even after passing stools, he used to feel as if he still had a lot of stool to pass. Patient’s blood pressure at the time of admission was 141/69 mmHg, heart rate of 108 beats per minute, respiratory rate of 28/min and temperature of 101.4 degree fahrenheit. Blood oxygen saturation was 95% on room air. Physical examination revealed slightly distended abdomen, however, rest of the physical examination, including neurological examination was completely unremarkable. Patient had good oral hygiene with no evidence of periodontal disease. Complete blood count revealed white blood cell count of 7.4 x 10^3 ^and hemoglobin of 12.1 g/dL. Urinalysis came out to be unremarkable for any evidence of infection. ESR was 32 mm/hour, whereas, serum CRP and pro-calcitonin were within normal limits. Sputum and blood culture sensitivity tests were ordered and the patient was empirically started on IV antibiotics (ceftriaxone). Two blood cultures grew *Parvimonas micra* and *Gamella morbillorum*. Infectious disease was consulted and they recommended switching the patient to ampicillin-sulbactam given the blood culture susceptibility results. Furthermore, they recommended doing CT scan chest/abdomen/pelvis and echocardiogram. There was no evidence of infective endocarditis on echocardiogram. However, CT scan abdomen/pelvis showed soft tissue mass in the ascending colon just superior to the ileocecal valve with shouldering suggesting an annular malignancy (fig.1,2). Patient’s fever and weakness started improving and infectious disease recommended to treat patient with at least 2 weeks of antibiotic duration. Gastroenterology was consulted and they performed colonoscopy which showed non-obstructing, eccentric, ulcerated, fungating, 4-5 cm diameter mass with necrotic areas (fig. 3). Biopsy of the mass revealed moderately differentiated adenocarcinoma. Given the fact that there was no evidence of distant metastasis, clinically and radiologically, oncology recommended surgical resection of the mass as definitive curative intervention. Also, patient was not deemed a candidate for systemic chemotherapy given his advanced age and comorbidities General surgery performed right hemicolectomy and removal of the mass. Pathology report of the mass revealed T3N0 and extension of the mass to the muscularis propria layer. At 2 weeks follow up visit with general surgery, patient was found free of constipation and fever.

## Discussion

*Parvimonas micra* is a gram-positive anaerobic cocci species (1). It is a part of the normal commensal flora of the gastrointestinal tract, and has been involved in infections of the periodontal area, soft tissue, bone and joints (2, 3). The conditions predisposing to anaerobic bacteremia including Parvimonas may involve malignant neoplasms, immune deficiencies, chronic renal insufficiency, decubitus ulcers, perforated abdominal viscus and appendicitis (4).

Our extensive literature search revealed that there has been only two case reports on bacteremia due to *Parvimonas micra*, one being in a patient with an esophageal tumour (1) and other being after (ERCP) endoscopic retrograde cholangiopancreatography (5). However, to our best knowledge, Parvimonas bacteremia in a patient with colonic carcinoma has never been reported before. Furthermore, there have been cases reported involving Parvimonas infection of different organs of the body, including, brain meninges (6), joints (3), heart valve (7), liver (8), vertebrae and intervertebral discs (9).

Anaerobic cocci are frequently isolated from the blood cultures in the setting of polymicrobial infections, therefore, their clinical role sometimes remains unknown (2). Of late, perhaps because of better microbiological diagnostic techniques 910) such as 16S rRNA gene sequencing or PCR assay (2, 11) and resultantly more documented cases of anaerobic bacteremia and anaerobic infections of the organs of the body, the clinical significance of anaerobic infections is becoming exceedingly important.

In addition to the above mentioned conditions predisposing to Parvimonas bacteremia, periodontal disease may also be another predisposing risk factor (1, 12). Unlike the above two referenced cases (1, 50 of Parvimonas bacteremia, our patient neither had periodontal disease nor underwent ERCP, respectively. However, he had colonic carcinoma, yet another time highlighting malignancy as a significant predisposing cause of anaerobic bacteremia. Other factors that might have predisposed our patient to anaerobic bacteremia could be old age and diabetes mellitus.

Because of the overall high mortality rate associated with anaerobic bacteremia, patients must be started on antibiotic therapy when the index of suspicion of anaerobic infection is high (13), especially, with the fact that Parvimonas micra is highly susceptible to antibiotics (1). This seems to be true in our case where the patient started improving within a few days of start of antibiotics. Moreover, from the aforementioned case description and discussion, it is reasonable to remark that the patients presenting with fever without any identifiable source of infection and with underlying predisposing risks factors for anaerobic bacteremia, empirical therapy with antibiotics may be started as soon as possible while awaiting for the definite blood culture results. Also, it will not be unreasonable to do work up for malignancy. Considering CT scan of the chest, abdomen, pelvis and endoscopy/colonoscopy to screen for occult gastrointestinal tract malignancy in its early stages while it is still localized would be very imperative, especially, with the fact that surgical resection is the only curative treatment modality for localized colon cancer.

In conclusion Immune deficiency is a risk factor for anaerobic bacteremia. Apart from starting patient on antibiotics without waiting for the results of blood culture and susceptibilities, a thorough search for malignancy should be done when a patient presents with anaerobic bacteremia, especially, when the source of infection is not known. This may potentially aid in identifying malignancy in earliest stages and, therefore, improving the treatment outcome.
